# Evaluating infection prevention and control implementation in hospitals of underdeveloped region of China using the standardized WHO-IPCAF tool

**DOI:** 10.3389/fpubh.2025.1749241

**Published:** 2026-01-12

**Authors:** Li Li, Kunkun Leng, Xuewei Du, Guilan Wang, Min Liu, Qinglan Meng

**Affiliations:** 1 Department of Hospital Infection Management, Public Health Department, Affiliated Hospital of Inner Mongolia Medical University, Hohhot, Inner Mongolia, China; 2 Institute of Disinfection and Vector Control, Hangzhou Center of Disease Control and Prevention, Hangzhou, Zhejiang, China; 3 Department of Infectious Diseases, Public Health Department, Affiliated Hospital of Inner Mongolia Medical University, Hohhot, China

**Keywords:** China, healthcare-associated infection, infection prevention and control, self-assessment, WHO-IPCAF tool

## Abstract

**Background:**

Healthcare-associated infections (HAIs) represent a major threat to patient safety worldwide. However, the implementation status of core infection prevention and control (IPC) measures in underdeveloped regions of mainland China remains understudied.

**Methods:**

Between June and August 2025, the Nosocomial Infection Control and Quality Improvement Center (NICQI) of the Inner Mongolia Autonomous Region conducted an online survey to evaluate IPC implementation in secondary and tertiary general hospitals within its jurisdiction. The survey employed the World Health Organization (WHO) Infection Prevention and Control Assessment Framework (IPCAF) to ensure a standardized and comprehensive assessment.

**Results:**

A total of 128 hospitals submitted valid questionnaires, yielding a response rate of 72.3%. The overall median IPCAF score across all hospitals was 620.0 (interquartile range [IQR]: 522.5–692.5). Median scores were 590.0 (IQR: 496.2–655.5) for secondary hospitals and 722.5 (IQR: 672.5–745.0) for tertiary hospitals, with a statistically significant difference between hospital grades (*p* < 0.001). Among the eight core components, the highest score was observed for “built environment, materials, and equipment” (CC8), with a median of 95.0 (IQR: 87.5–100.0); no significant difference was found across hospital grades for this component (*p* = 0.082). In contrast, the lowest score was for “IPC programs” (CC1), with a median of 62.5 (IQR: 44.4–80.0), which differed significantly between hospital grades (*p* < 0.001).

**Limitations:**

Approximately 30% of eligible hospitals did not participate in the survey, which may reflect inadequate prioritization of IPC or limited engagement. Additionally, the potential for social desirability bias exists, as some institutions may have overestimated their scores due to concerns about reputational impact. Furthermore, certain complex IPCAF concepts—such as multimodal strategies—may not have been fully understood by all respondents, possibly affecting the accuracy of the submitted data.

**Conclusion:**

While the overall IPCAF scores of secondary and tertiary hospitals in the Inner Mongolia Autonomous Region were relatively high, but more than half of the secondary hospitals demonstrated only intermediate or basic IPC implementation capabilities. This study elucidates the adoption status of the WHO-recommended IPC core components in a underdeveloped region of mainland China, thereby addressing a significant evidence gap in this field and providing a basis for targeted interventions and policy development.

## Background

Globally, the escalating threats posed by healthcare-associated infections (HAIs) and antimicrobial resistance (AMR) have become a top priority for ensuring patient safety in healthcare settings (1–3). Substantial evidence demonstrates that the effective implementation of evidence-based infection prevention and control (IPC) interventions is a crucial strategy for mitigating the risks associated with HAIs and AMR (4–6). The core components of IPC, as outlined by the World Health Organization (WHO), represent fundamental requirements for preventing the transmission of HAIs and AMR (7, 8). These components have been shown to reduce the incidence of HAIs by 35–70% (4, 9, 10).

To facilitate the development of IPC systems and the implementation of interventions at both national and healthcare facility levels, the WHO has delineated the core components essential for establishing robust IPC programs (4, 8). To assist healthcare facilities in systematically evaluating their IPC implementation status, the WHO introduced the IPC Assessment Framework (WHO-IPCAF) (11). This structured tool is designed for the comprehensive assessment of a healthcare facility’s IPC infrastructure and capacities. Developed in alignment with *the Guidelines on Core Components of Infection Prevention and Control Programmes* (8), the WHO-IPCAF includes questions pertaining to each core component outlined in these guidelines.

China is one of the largest developing countries in the world, characterized by its vast territory and uneven economic development. In comparison to more economically advanced regions such as the Yangtze River Delta, Pearl River Delta, Beijing-Tianjin-Hebei, and Shandong Peninsula, the Inner Mongolia Autonomous Region in northern mainland China is less economically developed. While several prosperous regions in China have already utilized the WHO-IPCAF to conduct relevant assessments, there is limited understanding of how hospitals in underdeveloped areas manage HAIs and control AMR. Specifically, there is a lack of information regarding the availability and implementation status of the IPC core components proposed by the WHO in these regions. Therefore, to contribute to the evidence base in this field, this study aims to use the standardized WHO-IPCAF tool (11) to assess the implementation of IPC in secondary and tertiary healthcare institutions within the Inner Mongolia Autonomous Region. This will illuminate the structure and current status of IPC in hospitals within this region.

This study aimed to assess the structure and current status of IPC in secondary and tertiary general hospitals within the Inner Mongolia Autonomous Region. Specialist hospitals, such as maternity and child health hospitals and stomatology hospitals, as well as traditional medicine hospitals, including Traditional Chinese Medicine and Mongolian Medicine hospitals, were excluded from the assessment.

## Methods

### Study design

This investigation was organized, implemented, data collected and analyzed by the Inner Mongolia Center for Nosocomial Infection Control and Quality Improvement (NICQI). It was a cross-sectional survey on the current status of IPC management conducted in Inner Mongolia Autonomous Region, China, from June to August 2025. The study adopted the standardized WHO-IPCAF tool. This investigation was jointly conducted by the NICQI of Inner Mongolia Autonomous Region and 12 regional NICQI centers within the jurisdiction, inviting all secondary and tertiary general hospitals within the jurisdiction to participate in this investigation. The purpose, methods and data collection of this study were detailedly introduced. The questionnaire was sent out through the existing NICQI management WeChat group.

### Study background

The Inner Mongolia Autonomous Region is located in the northern border of the Chinese mainland, presenting a long strip-shaped layout running from northeast to southwest. It has an east–west span of approximately 2,400 km, spanning three time zones, and the maximum north–south distance exceeds 1,700 km. The total area is 118,300 square kilometers, accounting for 12.30% of China’s land area, making it the third largest provincial administrative region in China. The total population of this region is 23.88 million, ranking 24th among all provincial administrative regions in China. According to the “*2019 China Regional Economic High-Quality Development Research Report*” (in Chinese) released by the China Regional Economic High-Quality Development Research Group, the Inner Mongolia Autonomous Region is classified as “medium-low quality zone.” In the “Economic Quality Gap Index” of this research report, the Inner Mongolia Autonomous Region scored 38 (out of 100), ranking 24th among the 31 provinces in the Chinese mainland^1^.

### Study participants

In the Chinese mainland’s Inner Mongolia Autonomous Region, currently there are 136 general hospitals classified as secondary and 34 hospitals classified as tertiary by the Inner Mongolia Autonomous Region Health Commission. This questionnaire survey invited all secondary and tertiary general hospitals within the jurisdiction to participate in a cross-sectional survey. Since the WHO-IPCAF tool assesses the hospital level rather than individual staff, respondents were allowed to ask or discuss with other colleagues if they were unsure of the answers. This was also done to ensure that the provided data was as reliable and accurate as possible. The online distribution of the questionnaire form was completed by the deadline, with a 2-week filling period. Therefore, respondents were given sufficient time to fill out the form. All participants gave informed consent before answering the questionnaire. This questionnaire form requires the full-time department heads of the IPC management departments of each hospital to fill it out; if there are no full-time personnel, then the heads of the hospital departments responsible for IPC activities should fill it out. This ensures the accuracy and uniqueness of the questionnaire filling, avoids duplicate submissions, and requires all participating institutions to submit the completed questionnaire forms before the end of this survey.

### Data collection

This online questionnaire survey is the first time that the WHO-IPCAF tool has been implemented in economically underdeveloped areas of the Chinese mainland. IPCAF is a structured closed-ended questionnaire, serving as a quantitative survey tool, and is accompanied by a corresponding scoring system. It covers eight core components (CCs): Infection Prevention and Control (IPC) plan (CC1), IPC guidelines (CC2), IPC education and training (CC3), healthcare-associated infections (HAIs) monitoring (CC4), multi-modal strategies for IPC intervention (CC5), IPC practice monitoring/audit and feedback (CC6), workload, staffing, and bed occupancy rate (CC7), and IPC building environment, materials, and equipment at the facility level (CC8). Based on the IPCAF score of the hospital, it is classified into four different IPC levels: insufficient (0–200 points), basic (201–400 points), intermediate (401–600 points), and advanced (601–800 points) (11). This study determined the IPC implementation levels of secondary and tertiary general hospitals in Inner Mongolia Autonomous Region by measuring the implementation levels of these eight core components and calculating the median score and interquartile range (IQR), thereby identifying the IPC implementation levels of these hospitals. The Inner Mongolia NICQI is responsible for translating IPCAF into a Chinese version and providing detailed instructions for filling out the questionnaire.

### Confidentiality and ethics

To accurately reflect the current status of IPC structures in hospitals across the region, we emphasized that participation in this survey was entirely voluntary and anonymous. Furthermore, we assured participants that IPCAF scores would not be included in the year-end performance assessments of hospital IPC work quality. To ensure the quality of the questionnaire responses, a dedicated hotline was established, with personnel assigned specifically to address any queries. This project received approval from the Biomedical Research Ethics Committee of Inner Mongolia Medical University (No: YKD202401076).

### Statistical analysis

Descriptive analysis of the data was performed using Excel. The IPCAF scores were summarized by calculating the medians and interquartile ranges (IQR). Additionally, median and IQR values for the IPCAF scores were computed. All data analyses were conducted using R version 4.5.1. The Shapiro–Wilk test and Levene’s test were employed to assess the normality of the data and the homogeneity of variances, respectively. Results for quantitative variables are presented as median (*P*_25_, *P*_75_). The Wilcoxon Rank-Sum Test, a non-parametric method, was utilized to compare differences between two groups. All statistical tests were two-sided, and a *p*-value of less than 0.05 was considered statistically significant.

## Results

### Characteristics of participating hospitals

In this online survey, 128 general hospitals submitted valid questionnaires. Among these, 95 were secondary hospitals (response rate: 69.9%, 95/136) and 33 were tertiary hospitals (97.1%, 33/34). Of the 128 hospitals, 9 (7.0%) had fewer than 100 beds; 81 (63.3%) had 100–499 beds; 22 (17.2%) had 500–999 beds; and 16 (12.5%) had 1,000 or more beds. All questionnaires were completed by the heads of dedicated IPC management departments at each institution. Where no such department existed, the heads of relevant departments responsible for IPC management completed the questionnaire.

### IPCAF score distribution

**Figure 1:**
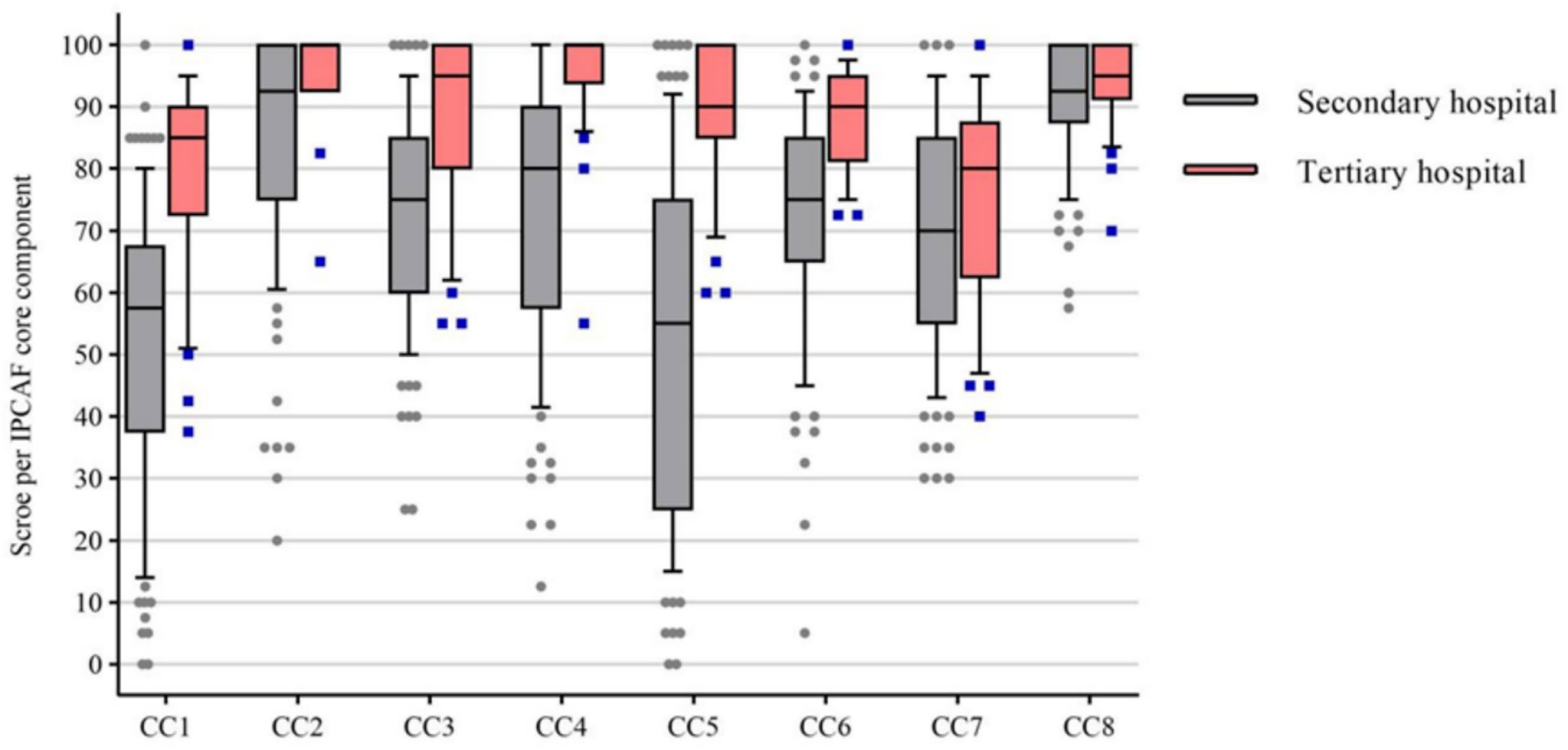
Distribution of scores for the eight core components (CC). Each CC has a maximum score of 100. Box plot illustrating the overall median, interquartile range, and outliers for each CC.

The overall median IPCAF score across the 128 general hospitals was 620.0 (IQR: 522.5–692.5). The median score for secondary hospitals was 590.0 (IQR: 496.2–655.5), while that for tertiary hospitals was 722.5 (IQR: 672.5–745.0). A statistically significant difference in scores was observed between hospital grade (*p* < 0.001). Among the eight core components, the component related to building environment, materials, and equipment (CC8) had the highest overall median score of 95.0 (IQR: 87.5–100.0), with no significant difference between secondary and tertiary hospitals (*p* = 0.082). In contrast, the IPC program component (CC1) had the lowest overall median score of 62.5 (IQR: 44.4–80.0), and scores differed significantly between secondary and tertiary hospitals (*p* < 0.001) (Table 1 and Figure 1).

**Table 1:** Distribution of overall IPCAF scores and scores by core component among 128 general hospitals in the Inner Mongolia Autonomous Region.

Core component (CC)	Secondary hospital median (*P*_25_, *P*_75_)	Tertiary hospital median (*P*_25_, *P*_75_)	Overall median (*P*_25_, *P*_75_)	*p*-value
CC1	57.5(38.8,67.5)	85.0(72.5,90.0)	62.5(44.4,80.0)	<0.001
CC2	92.5(75.0,100.0)	100.0(92.5,100.0)	92.5(82.5,100.0)	<0.001
CC3	75.0(62.5,85.0)	95.0(80.0,100.0)	80.0(65.0,90.0)	<0.001
CC4	80.0(58.8,90.0)	100.0(95.0,100.0)	85.0(70.0,95.0)	<0.001
CC5	55.0(25.0,75.0)	90.0(85.0,100.0)	65.0(35.0,90.0)	<0.001
CC6	75.0(65.0,85.0)	90.0(82.5,95.0)	80.0(67.5,90.0)	<0.001
CC7	70.0(55.0,82.5)	80.0(65.0,85.0)	70.0(55.0,85.0)	0.147
CC8	92.5(87.5,100.0)	95.0(92.5,100.0)	95.0(87.5,100.0)	0.082
Overall median (*P*_25_, *P*_75_)	590.0(496.2,655.5)	722.5(672.5,745.0)	620.0(522.5,691.2)	<0.001

CC1 = Infection Prevention and Control (IPC) plan; CC2 = IPC guidelines; CC3 = IPC education and training; CC4 = Healthcare-associated infections (HAIs) monitoring; CC5 = Multi-modal strategies for IPC intervention; CC6 = IPC practice monitoring/audit and feedback; CC7 = Workload, staffing, and bed occupancy rate; CC8 = IPC building environment, materials, and equipment at the facility level.

**Figure 2:**
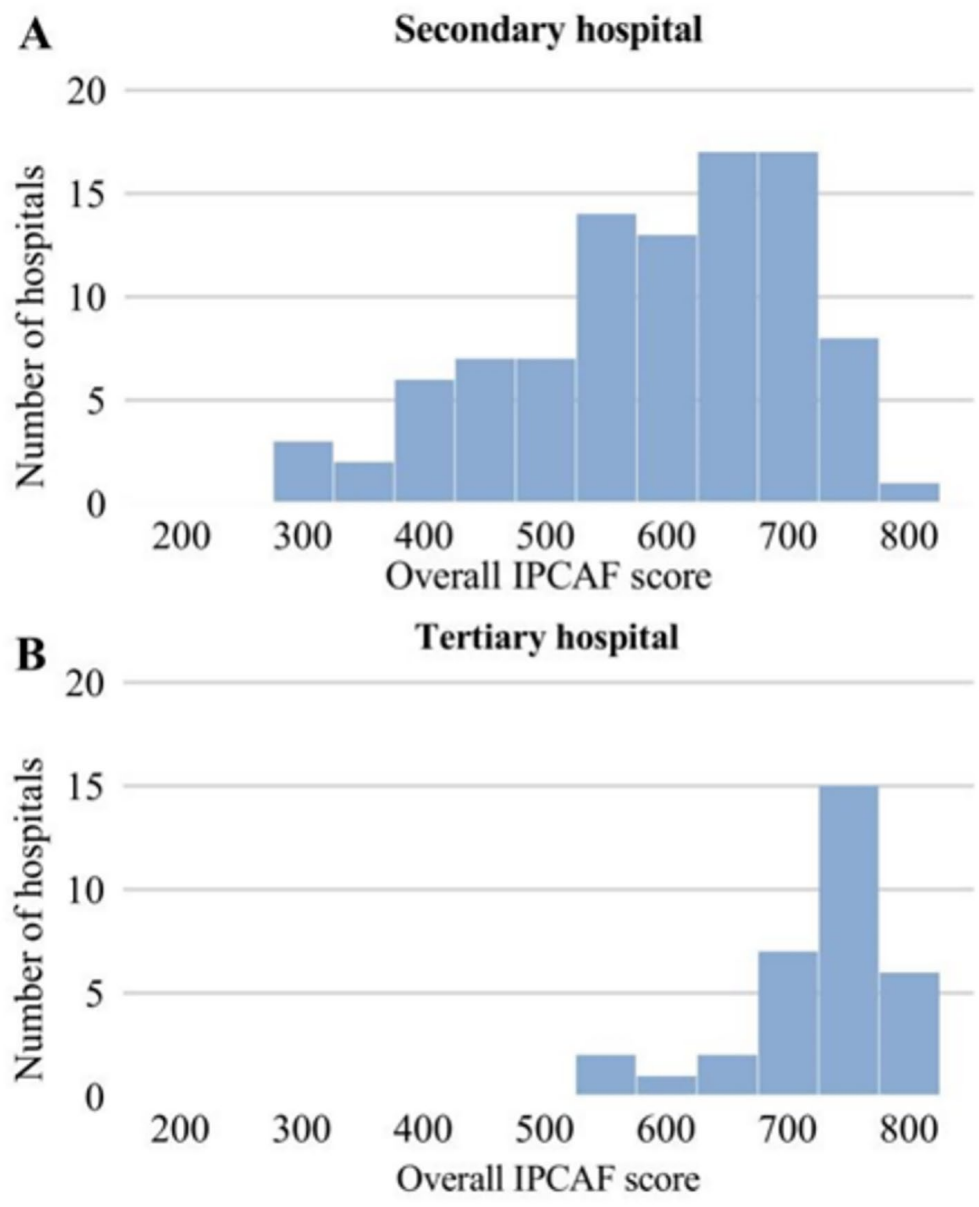
Distribution of IPCAF scores among general hospitals in the Inner Mongolia Autonomous Region. **(A)** Indicates secondary general hospitals; **(B)** indicates tertiary general hospitals. IPCAF, infection prevention and control assessment framework.

According to the WHO-IPCAF classification, among secondary general hospitals, 11 (11.6%) were rated as basic, 40 (42.1%) as intermediate, and 44 (46.3%) as advanced. Among tertiary general hospitals, 3 (9.09%) were rated as intermediate and 30 (90.91%) as advanced. Notably, no hospitals were classified as insufficient (Table 2 and Figure 2). The distribution of IPC management levels differed significantly between hospital grades (*p* < 0.001).

**Table 2:** Classification of IPC implementation levels in secondary and tertiary general hospitals in Inner Mongolia Autonomous Region.

Classification	Score range	Secondary hospital *n* (%)	Tertiary hospital *n* (%)
Insufficient	0–200	Zero	Zero
Basic	201–400	11 (11.58)	Zero
Intermediate	401–600	40 (42.11)	3 (9.09)
Advanced	601–800	44 (46.31)	30 (90.91)
Total		95 (100.00)	33 (100.00)

### IPC program

The overall median score for IPC planning (CC1) was notably low at 62.5 (IQR: 44.4–80.0), with a significant difference between secondary and tertiary general hospitals (*p* < 0.001). An independent IPC management department was established in 121 hospitals (94.5%), while IPC management was under nursing or other administrative departments in 7 hospitals (5.5%). Most hospitals had formed hospital infection management committees, although 16 (12.5%) lacked a hospital-level committee. Most hospitals (*n* = 115) had set IPC work objectives; however, only 30 (23.4%) quantified IPC indicators and future targets, and only 29 (22.7%) had a dedicated IPC management budget.

### IPC guidelines

Among the 128 general hospitals, 81 (63.3%) had the capacity to develop or revise IPC guidelines. The majority (92.2%) had established IPC management systems for multidrug-resistant bacteria, 119 (93.0%) had systems for hospital-acquired pneumonia, and 120 (93.8%) implemented IPC management for catheter-related bloodstream infections. Additionally, 120 hospitals (93.8%) conducted routine monitoring of HAIs to assess IPC effectiveness.

### IPC education and training

Among the participating hospitals, 83 (64.6%) had non-IPC professionals with sufficient skills acting as trainers or mentors. Only 39 hospitals (30.5%) used traditional lecture-based training, whereas 89 (69.5%) employed interactive training methods for healthcare workers. Nearly all hospitals evaluated the effectiveness of their training programs. Furthermore, 77 hospitals (60.2%) provided specific IPC-related training to patients or their families.

### HAIs monitoring

Almost all hospitals (*n* = 124) considered HAIs monitoring a key component of IPC management. Professionally trained personnel were available in 117 hospitals (91.4%), and 103 (80.5%) utilized IT support for HAIs monitoring and data processing. Device-related infections were monitored in 122 hospitals (95.3%), multidrug-resistant bacterial infections in 96 (75.0%), and surgical site infections (SSIs) in 122 (95.3%). However, only 95 hospitals (74.2%) assessed the correlation between HAIs monitoring data and IPC implementation. While 78.1% of hospitals had basic microbial identification capabilities, only 80 (62.5%) could simultaneously identify microbial species and their resistance patterns.

### Multimodal strategy implementation

This component received the lowest median score among the eight core components, at 65.0 (IQR: 35.0–90.0) (Table 1). Only 83 hospitals (64.8%) in the region adopted a multimodal strategy for IPC interventions, and merely 60.9% (*n* = 78) used interdisciplinary teams in IPC work. Systematic corrective measures were implemented in 102 hospitals (79.7%), and 120 (93.8%) provided feedback on HAIs monitoring data. However, communication and reminders to relevant departments or personnel were conducted in only 71.1% (*n* = 91) of hospitals, and only 75 (58.6%) promoted a patient safety culture within their institutions.

### Monitoring/audit and feedback

A total of 106 hospitals (82.8%) had personnel trained to monitor, audit, and provide feedback on IPC practices. Regarding monitoring processes, only 60 hospitals (46.9%) monitored wound dressing procedures. Preventive measures based on transmission and isolation to control multidrug-resistant organisms (MDROs) were implemented in 101 hospitals (78.9%). Consumption of hand hygiene products was tracked in 99 hospitals (77.3%). However, only 45 hospitals (35.2%) conducted at least one annual self-assessment using the WHO hand hygiene self-assessment framework, and only 27 (21.1%) used survey tools to assess factors influencing hospital safety culture.

### Workload, staffing allocation and bed occupancy rate

Only 54 hospitals (42.2%) reported assessing staffing levels based on patient numbers and in accordance with national standards or tools such as the WHO Workload Indicator Staffing Needs method. Moreover, only 24 (18.8%) reported full compliance with staffing standards throughout the hospital. While most hospitals (89.8%) did not add beds in corridors, only 85 (66.4%) had assessment and response mechanisms for excessive bed occupancy.

### Building environment, materials and equipment

The overall median score for CC8 was the highest among the eight components, at 95.0 (IQR: 87.5–100.0), with no significant difference between secondary and tertiary hospitals (*p* = 0.082) (Table 1). All 128 hospitals reported a continuous and adequate water supply meeting all needs, including reliable drinking water for healthcare workers, patients, and families in all wards. Functional hand hygiene facilities and products were available at all care points with sustained supply. Effective environmental ventilation systems in patient care areas were reported by 123 hospitals (96.1%).

Finally, it should be noted that during the survey period, our hotline received approximately 30 inquiries. Most related to clarification of text descriptions, particularly regarding overlap between the multimodal strategy content and other core components. We clarified that implementation of three or more of the five technospheres of the multimodal strategy was required for it to be considered a multimodal intervention practice.

## Discussion

To the best of our knowledge, this study represents the first large-scale assessment of the structure and current status of IPC in healthcare facilities located in an underdeveloped region of mainland China, utilizing the WHO-IPCAF. A total of 128 general hospitals participated in the survey, which included 95 secondary hospitals (69.9%, 95/136) and 33 tertiary hospitals (97.1%, 33/34). This participation ensures strong representativeness and provides a robust dataset for understanding the current state of IPC practices in an underdeveloped region of China. The findings revealed that the overall median score on the WHO-IPCAF for secondary and tertiary general hospitals in the Inner Mongolia Autonomous Region was 620.0. Specifically, the total median score for secondary hospitals was 590.0 (IQR: 496.2–655.5), while for tertiary hospitals, it was 722.5 (IQR: 672.5–745.0). There was a statistically significant difference in scores among hospitals of different grades (*p* < 0.001).

Based on a comparison with global IPCAF survey literature, the total median score of the IPCAF in the Inner Mongolia Autonomous Region is classified as “Intermediate.” Notably, this score is significantly lower than those of Germany (12, 13), Austria (14), Turkey (15), Japan (16), and Tehran, Iran (17) in the earlier years. Additionally, this score was lower than that of Zhejiang Province in China mainland as reported in 2022 (18) and that of Shaanxi Province as reported in 2025 (19). In contrast, the IPCAF score for Inner Mongolia is comparable to that of India (20) and Indonesia (21), both of which have an overall median IPCAF score of 620.0. However, it is higher than the scores reported in Latin America (22), several African countries (23–27), Pakistan (28), Bangladesh (29), and Punjab, Pakistan (30). A questionnaire survey that collected 4,440 responses on IPC implementation status from 81 countries across six WHO regions indicated that IPCAF levels vary according to regional economic income levels (31). The IPC structure and capacity level obtained in this study were higher than those in low-and middle-income countries (500.4; *n* = 711), but lower than those in high-middle-income countries (632.5; *n* = 1,511) (31).

Based on the cross-sectional survey of the IPC structure and capacity building of secondary and tertiary general hospitals in Inner Mongolia Autonomous Region, it was found that the secondary and tertiary hospitals in our region are at the intermediate level as classified by WHO. Particularly, in the eight core components, the scores for “building environment, materials, and equipment (CC8)” and “IPC guidelines (CC2)” were the highest, demonstrating outstanding performance. This is mainly attributed to the fact that in recent years, especially after the fight against the COVID-19 pandemic, the local health administrative department has increased investment in the basic infrastructure and equipment for IPC. Additionally, the Inner Mongolia Autonomous Region NICQI conducts routine supervision and inspections of the implementation status of HAIs management in all hospitals within the region in various forms (such as written reports and on-site inspections) every year. IPC guidelines, as one of the important inspection items, has received attention from all hospitals, thereby gradually improving the formulation of IPC guidelines in all hospitals.

Among the eight core components, the overall median score for the IPC program (CC1) in hospitals within our region was the lowest, at 62.5, revealing a statistically significant difference between secondary and tertiary hospitals (*p* < 0.001). From a national perspective, IPC management in China began later than in European and American countries. In 1986, the Medical Administration Department of the former Ministry of Health organized the first national symposium on hospital infections and established a coordination group for hospital infection surveillance and research, along with a national hospital infection surveillance network. This marked the formal initiation of IPC management in China (18, 19). Subsequently, Nosocomial Infection Control and Quality Improvement (NICQI) programs were established at the provincial level across various provinces during the 1990s; however, the Inner Mongolia Autonomous Region did not establish its NICQI until 2005. This delay indicates that IPC management in our region started later than in other provinces, leading to relatively limited capacity building in IPC. The findings of this survey revealed that only 30 hospitals (23.4%) had quantified IPC work indicators and future goals; an additional 37 hospitals (28.9%) had quantified IPC indicators but lacked defined future goals. Among the 128 general hospitals surveyed, only 29 (22.7%) allocated budgetary resources for IPC work. Although the majority of hospitals in our region (87.5%) have established hospital infection control committees and have independent infection control management departments (94.5%), effective infection control can only be achieved by formulating comprehensive infection control plans, providing sufficient personnel, allocating adequate budget resources, and obtaining support from hospital management (32). Therefore, we will use multiple approaches and forms, and present our information in written reports to the local health administrative department, requesting an increase in IPC special funds for all hospitals at all grades; at the same time, we plan to conduct a national survey to compare the gap in IPC special funds between Inner Mongolia Autonomous Region and other provinces in China, as well as to suggest the necessity of increasing special IPC funds for risk control of HAIs, and form a special survey report to submit to the local health administrative department. While our region boasts a relatively high percentage of hospitals with established Infection Control Committees, there is clearly inadequate support for IPC management departments in executing their practical responsibilities. This is particularly evident in the significant lack of financial investment in IPC initiatives, which has impeded the rapid development of IPC management in hospitals throughout our region.

The core of the IPC multimodal implementation strategy is to support the translation of evidence and guideline recommendations into healthcare practices, thereby changing the behavior of healthcare workers (4). Numerous studies have demonstrated that adopting multimodal strategies significantly reduces HAIs and improves hand hygiene compliance. For example, multimodal interventions have shown notable success in various research fields, including enhancing hand hygiene adherence (33, 34), preventing central line-associated bloodstream infections (35, 36), reducing surgical site infections (37, 38), curbing the spread of drug-resistant bacteria (39, 40), and preventing hemodialysis-related infections (41). However, existing literature indicates that the implementation of IPC multimodal strategies is generally suboptimal (12, 14–16, 18). Findings from this survey reveal that, among the five multimodal strategy areas recommended by the WHO, the least satisfactory outcomes are observed in two key aspects: only 75 hospitals (58.6%) have fostered a patient safety culture throughout the hospital, and merely 71.1% (*n* = 91) of hospitals communicate and provide reminders about HAIs surveillance data to relevant departments or personnel. Therefore, hospitals at all levels must introduce or adopt multimodal intervention strategies in their future IPC activities to enhance nursing practices, reduce the incidence of HAIs, and combat AMR, which poses the greatest challenge for hospitals in our region.

A scientifically sound workload, appropriate staffing levels, and optimal bed occupancy rates are essential not only for reducing the risk of HAIs and the spread of AMR but also for delivering high-quality health services within the framework of universal health coverage (32). However, the findings of this survey reveal that, when assessing staffing levels in secondary and tertiary hospitals in the Inner Mongolia Autonomous Region using national standards or WHO assessment tools such as the “*Workload Indicators of Staffing Need*” method, only 54 hospitals (42.2%) reported conducting such evaluations. Moreover, only 24 hospitals (18.8%) indicated that the ratio of healthcare workers to patients throughout the hospital met national or WHO standards. Regarding compliance with national or international standards for ward designs based on the number of beds, only 64 hospitals (50.0%) reported meeting the criteria across the entire facility. Clearly, local health administrative departments need to increase investments in human resources and allocate healthcare workers more rationally based on patient workload.

This study has several limitations. First, 30% of medical institutions in the region did not participate in the survey. This non-participation may have stemmed from some hospitals’ insufficient attention to IPC, which could have led to a lack of enthusiasm for participation. Additionally, despite our pre-survey statement assuring that IPCAF scores would not influence hospital performance evaluations, some institutions remained concerned that low scores might adversely affect their reputation. Therefore, the IPCAF scores in this study may be overestimated, which implies that the research results may not fully represent the hospital of IPC in the hospitals of our region. Second, certain complex IPCAF concepts—such as multimodal strategies—may not have been fully understood by all respondents, possibly affecting the accuracy of the submitted data. Third, although explanatory footnotes were provided in the survey, not all participants may have been familiar with certain complex concepts mentioned in the IPCAF, such as multimodal strategies, potentially leading to inaccurate responses. Fourth, some questions may have been perceived as sensitive by participants, and despite the survey’s assurance of confidentiality, biased responses could still have been elicited.

In summary, this study only focused on the secondary and tertiary general hospitals within the Inner Mongolia Autonomous Region of the Chinese mainland, and did not include specialized hospitals or traditional medical hospitals. Therefore, it is necessary to be extremely cautious when extending the conclusions in this study to all underdeveloped areas or other low-resource environments across the Chinese mainland. Furthermore, it is necessary to clearly state that the content of this questionnaire survey does not include any data related to the incidence rates of HAIs in each hospital. Therefore, this study was unable to assess whether higher IPCAF scores translate into better clinical outcomes.

## Conclusion

Although secondary and tertiary general hospitals in the Inner Mongolia Autonomous Region achieved a relatively high overall IPCAF score, significant disparities in IPC implementation and core components exist among hospitals of different grades. It is worth noting that this survey reveals that more than half of the secondary general hospitals are still at a intermediate or basic level. Despite potential differences in legal, policy, and regulatory environments, these guidelines are relevant for both developed and underdeveloped countries/regions, as the need for effective IPC programs is universal across various cultures and contexts. In underdeveloped countries/regions, there is a particular need for local governments to invest in funding and human resources, carefully evaluate the feasibility and cost-effectiveness of IPC implementation, and promote the comprehensive application of WHO IPC core components through rational strategies and practical tools, thus maximizing patient safety benefits. This study highlights the current status and implementation levels of WHO-recommended IPC core components in an underdeveloped region of mainland China, addressing a significant research gap in this research field.

## Data Availability

The original contributions presented in the study are included in the article/supplementary material, further inquiries can be directed to the corresponding author.

## Footnotes

1
http://paper.ce.cn/jjrb/page/1/2020-01/22/10/2020012210_pdf.pdf
